# Health Professionals’ Chronotype Association with Salivary Cortisol and Occupational Stress in Neonatal Intensive Care Units

**DOI:** 10.3390/ijerph20095683

**Published:** 2023-04-28

**Authors:** Jocélia Maria de Azevedo Bringel, Isabel Abreu, Maria-Cláudia Mendes Caminha Muniz, Paulo César de Almeida, Maria-Raquel G. Silva

**Affiliations:** 1 Faculty of Science and Technology, University Fernando Pessoa, 4249-004 Porto, Portugal; 2 FP-I3ID, University Fernando Pessoa, 4249-004 Porto, Portugal; 3 Postgraduate Program in Neuropsychology, Universidade Christus, Fortaleza 60160-230, Brazil; fgaclaudia10@gmail.com; 4 Postgraduate Program in Clinical Health Care Nursing, Universidade Estadual do Ceará, Fortaleza 60714-903, Brazil; 5 Faculty of Health Sciences, University Fernando Pessoa, 4200-150 Porto, Portugal; 6 CIAS-Research Centre for Anthropology and Health—Human Biology, Health and Society, University of Coimbra, 3000-456 Coimbra, Portugal; 7 CHRC-Comprehensive Health Research Centre-Nova Medical School, Nova University of Lisbon, 1150-090 Lisbon, Portugal

**Keywords:** cortisol, health personnel, work-related stress, chronobiology phenomena, neonatal intensive care

## Abstract

Burnout syndrome has been reported among health workers, particularly those working in critical areas, and is considered a significant public health problem. This study aimed to investigate the relationship between chronotype and work-related stress, as measured by salivary cortisol levels and burnout, among health professionals working in neonatal intensive care units. A cross-sectional study was conducted across four public hospitals in Fortaleza, Ceará, Brazil. Two hundred and fifty-six health professionals were administered the brazilian version of the Burnout Characterization Scale, the morningness–eveningness questionnaire, for chronotype, a sociodemographic questionnaire that included lifestyle habits and a salivary cortisol test. The results indicated that morning chronotype workers were significantly associated with the following: advanced age (*p* < 0.001), female gender (*p* = 0.032), married status (*p* = 0.014), and having children (*p* = 0.030) compared to those with evening and intermediate chronotypes. However, no significant association was found between signs of burnout syndrome and chronotype (*p* = 0.316). Participants whose work shift did not match their chronotype had significantly higher initial salivary cortisol levels (*p* = 0.013). The findings suggest that adapting working hours to an individual’s biological rhythm can help mitigate potential negative effects on physical and mental health. Thus, it is recommended that professionals’ working hours be adjusted accordingly.

## 1. Introduction

Working in intensive care units is one of the most stressful occupational activities, especially for nursing professionals, as it requires high levels of attention, concentration, and reactivity [1]. Continuous demands and complex activities enhanced by inadequate working conditions (noise from machines’ alarms, overcrowding [2,3], long working hours, and severe conditions of patients) [4] have been associated with high level of stress in healthcare professionals. This can negatively affect their physical and mental health [5] as well as impairing patient care [6], increasing the risk of errors and unfavorable outcomes [7].

Work-related stress is defined as an imbalance between the required workload and the worker’s ability to respond accordingly, causing negative changes in both the emotional and physical states of the professional [8]. In addition, exposure to prolonged stressful situations at work can lead to the occurrence of Burnout Syndrome, which is characterized by emotional exhaustion, depersonalization, and reduced personal fulfillment [9].

Furthermore, high levels of anxiety (caused by constantly seeing others in pain, experiencing tragedies related to patients’ deaths, depression, and illness) can generate physical, psychological, and psychomotor changes that vary in intensity according to the resistance of each individual, often leading to a low perception of burnout [10].

In recent years, the high incidence of burnout syndrome in health workers, especially in those working in more critical areas, has been considered a public health problem [4]. This has been especially related to the increase in absenteeism levels at work [1,11] causing overload for other professionals, negative impacts on the provision of services, reductions in productivity, low incomes, the rise in financial costs, and a reduction in the quality of healthcare [12]. In fact, the latter has been reported regarding stressful doctor–nurse interactions and the occurrence of burnout [6,7,13,14]. Although most studies report that nurses are the group at most risk for developing Burnout Syndrome [15], it can affect any professional, namely physicians [16,17] who have been recently overwhelmed because of the COVID-19 pandemic [18].

Existing studies show that this syndrome is caused by the increase of cortisol secretion [19] when exposed to prolonged stressful situations, including working activities or even being unemployed [20,21,22]. Cortisol is a steroid hormone produced by the adrenal cortex, which regulates a wide range of vital processes throughout the body, including metabolism and immune response. Physiologically, it presents a circadian variation with a maximum level in the morning and decreasing throughout the day to reach a minimum value around midnight [23]. Stressful situations occurring chronically can lead to an imbalance in this homeostasis [24].

In response to stressful situations, the human body reacts by activating its regulatory systems, namely the adrenal sympathetic axis (ASA) and the hypothalamic pituitary adrenal (HPA) axis. There is an immediate response to the offending agent, which is triggered by the activation of the ASA, which releases so-called catecholamines into the bloodstream, raising the heart rate and blood pressure, giving the individual the necessary strength to escape in adverse situations. On the other hand, the HPA axis triggers a slower response from the body, activating communication between the brain and the endocrine system through the production of glucocorticoids by the cortex of the adrenal gland, with cortisol being released [25,26].

Salivary cortisol levels have been used as an important biomarker to objectively assess the functioning of the HPA axis in situations that can lead to cognitive changes, such as in times of stress and anxiety [27]. In fact, cortisol levels found in saliva are similar to those found in plasma, being easily available in one’s saliva between two and three min after stressful situations occur [28,29,30]. In addition, the low cost, simplicity of collection and transport, and the stability of samples contribute to the adherence of participants; as a result, salivary cortisol levels are widely used in scientific research.

In addition to the circadian variation in cortisol, the human body works in cycles controlled by a 24 h period, known as circadian rhythm, and under normal conditions, it follows a day–night cycle [31,32]. This biological rhythm of the central nervous system is determined by an individual’s genetics, health conditions, and environmental factors (including his/her occupation/job) [33] and is responsible for the chronotype differences observed in relation with individuals’ preferences to carry out daily activities more in the morning, afternoon, or evening.

Chronotypes or circadian typology are the individual variations in biological and behavioral patterns, determining each individual’s phases of higher mental and physical performance. It is a functional state of the individual [34,35,36]. 

We can classify individuals according to their preferences for carrying out certain activities into three types of chronotypes: morning, evening, and intermediate. People with a morning chronotype prefer going to bed early and waking up early; people with an evening chronotype prefer going to bed late and waking up late. The intermediate chronotype falls between these two extremes [35,37].

Some activities, such as those performed by health professionals working in shifts, demand from the individual a desynchronization of their biological sleep–wake pattern, leading to negative effects, such as sleep disorders, reduced professional performance, and psychiatric disorders, which impact the individual’s quality of life [38,39]. Knowing the types of chronotypes for these professionals is important for adapting their activities, generating better professional performance and an increased quality of life [38].

This study aims to evaluate occupational stress and salivary cortisol in health professionals working in neonatal intensive care units (NICU) and test for any evidence of an association with chronotype. The following research questions are proposed: What is the predominant chronotype among professionals working in the neonatal intensive care unit? Are these professionals performing activities in synchrony with their chronotype? Is there any association between health professionals’ chronotypes, cortisol measurements, and stress levels?

The correlation between chronotype and stress has been little studied in the literature. This study hopes to contribute to improving the quality of life of health professionals who work in neonatal intensive care, understanding the correlation between their chronotype, stress level, and quality of life.

## 2. Materials and Methods

### 2.1. Study Design

This is a cross-sectional, quantitative study with a convenience sample. A sample consisting of 256 health professionals who worked in the neonatal intensive care unit of four public hospitals (General Hospital Dr. Waldemar Alcântara, Hospital Geral de Fortaleza, Hospital Geral Dr. César Cals, and Maternidade Escola Assis Chateaubriand) in Fortaleza, Ceará, Brazil.

### 2.2. Participants

A total of 256 individuals were selected for this study.

The inclusion criteria were having worked in an NICU for at least for 6 months; and being able to fulfil the prerequisites for giving the necessary biological material for the study.

Exclusion criteria were not completing the questionnaires, not collecting both cortisol samples at the beginning and/or at the end of one’s shift and being treated with corticosteroids.

All participants gave their informed consent to take part in this study.

### 2.3. Procedure

Data collection took place between June 2019 and November 2020 on four to five consecutive weekdays, which corresponded to the period with greater urgencies flow, and covered all work shifts. Initially, the head of each NICU was contacted and, after consenting to carry out the research, health professionals were contacted and received information on the study objectives and all procedures.

All the participants answered a questionnaire consisting of four parts: the first part was sociodemographic work conditions and lifestyle habits, including specific information to be investigated as prepared by the authors; the second part was the Burnout Characterization Scale; the third part was the morningness–eveningness questionnaire (MEQ-SA) by Horne and Östberg for chronotype classification; the fourth part was information about work conditions, anthropometric indicators, and perceptions of tiredness and stress before and after work shifts.

The salivary cortisol dosage was collected at the beginning and end of work shifts.

#### 2.3.1. Sociodemographic Information, Work Conditions, and Lifestyle Habits

Several parameters were obtained, namely age, sex, marital status, education level, household members, and lifestyle habits. Professional data included their position/function in the hospital, working schedule, shifts, time spent commuting, and potential incidents while working. Diseases, the use of any medication, physical activity habits, smoking, and alcohol consumption were also assessed. This part was prepared by authors.

#### 2.3.2. Burnout Characterization Scale

To assess the participants’ subjective stress, a questionnaire was conducted, inspired by the Maslach Burnout Inventory (MBI) which is recognized as a reliable measure of burnout with a Cronbach’s alpha coefficient above 0.70. It was translated to Portuguese and validated by Tamayo and Tróccoli in 2009. It comprises 20 questions with a score system for answers ranging from 1 to 5, which indicate the frequency that individuals experience the content indicated by the item, listed as follows: 1—Never, 2—Annually, 3—Monthly, 4—Weekly, 5—Daily. It evaluates three components: emotional exhaustion, depersonalization, and decreased professional fulfillment.

The questionnaire was answered by the interviewees with the objective of investigating psychophysical characteristics in relation to work, in order to obtain a score that could indicate the occurrence and degree of Burnout Syndrome, in which the sum of the points represents: from zero to 20 points: “no signs of Burnout”, from 21 to 40 points: “possible Burnout”, from 41 to 60 points: “initial phase of Burnout”, from 61 to 80 points: “Installed Burnout”, and from 81 to 100 points: “considerable phase of Burnout”.

#### 2.3.3. Morningness–Eveningness Questionnaire

The participants’ chronotype was assessed using the validated version of the morningness–eveningness questionnaire (MEQ-SA) by Horne and Östberg in Portuguese, which has been recognized as a reliable instrument to determinate chronotype, with a Cronbach’s alpha coefficient above 0.70, and has been widely used in the literature [40]. It is made up of 19 questions to which scores are assigned and together classify the individual’s chronotype into three types: morning (above 59 points), evening (less than 41 points), or intermediate (between 42 and 58 points). It also designates a category called “chronotype unfavorable” for participants whose work schedule is not aligned with their chronotype, which is morning-type workers working on night shifts and evening-type workers working day/morning shifts. In addition, work shifts were categorized as: 6 h morning shifts, 6 h afternoon shifts, 12 h daytime shifts, and 12 h nighttime shifts, according to the hospital’s organization.

#### 2.3.4. Salivary Cortisol

The participants collected their salivary cortisol at the beginning and at end of their work shifts under the supervision of the researcher using Salivette^®^ tubes with a roll made of synthetic fibers by Sarstedt [41]. Salivary cortisol dosage was requested twice: the first collection time was up to 1 h after the start of one’s shift, and the second collection time was up to half an hour before and after the end of one’s shift. Samples were collected in the participants’ work environment and placed in thermal boxes at temperatures between 2 °C and 8 °C. Immediately after, they were centrifuged in a Centrifuge Excelsa^®^ II (Model 206-BL, manufactured by FANEM, Brazil) for 2 min at a speed of 2000 rpm and kept refrigerated between 2 °C and 8 °C. They were then sent to the Clinical Diagnostic Center of Brazil—BIOSLAG LTDA, located in Fortaleza, Ceará, Brazil, to assess the dosages. The chemiluminescence method was used for the analysis due to its reliability and precision [28,39,40,41,42,43], and results were presented in micrograms per deciliter (µg/dL). Salivary cortisol values considered normal in comparison with those in the literature were as follows: between 6 a.m. and 10 a.m. they should be less than 0.736 µg/dL; and between 4 p.m. and 8 p.m. they are expected to be less than 0.252 µg/dL [44]. To enable group comparison and minimize bias, we classified each individual as either “normal” or “altered” based on the reference values for each time of saliva sample collection. All “altered” values were found to be higher than the baseline values.

All participants were informed about precautions prior to the salivary cortisol collection, such as not drinking alcohol, not smoking, and a 2 h interval between the biological collection and food intake or brushing their teeth.

Four additional questions were included regarding the health professionals’ perception of their state at the beginning and end of their work shift—very tired, not very tired, or rested; events that could have changed their work routine; and whether their work shift was “tiring” and/or “stressful”.

#### 2.3.5. Anthropometric Profile

Body mass and height were reported by participants. Body mass index (BMI) was calculated as a ratio of weight to the squared height (kg/m^2^). BMI was classified according to the following categories: normal (18.5–24.9 kg/m^2^), overweight (25.0–29.9 kg/m^2^), and obese (≥30.0 kg/m^2^) [45,46].

#### 2.3.6. Statistical Analysis

For statistical analysis, SPSS software for Macintosh, version 23 (IBM Corp.: Armonk, NY, USA) was used. Categorical variables were expressed as absolute counts and percentages and were compared using the Chi-square test or Fisher’s exact test. All quantitative variables were initially assessed for normality using the Shapiro–Wilk test and for data asymmetry through standard deviation, histogram analysis, and QQ diagrams. Data considered normal were then expressed as mean and standard deviation, and those considered non-normal were expressed as median and interquartile (IQ) range. For comparison between two groups, the Student’s *t* test or the Mann–Whitney test were used for normal and non-normal data, respectively. In the comparisons of three groups, the ANOVA test was used through the Tukey‘s post hoc test or the Kruskal–Wallis’s test with Dunn’s post hoc test for normal and non-normal data, respectively. Statistical significance was established as *p* < 0.05.

### 2.4. Ethical Approval

This study was submitted to Plataforma Brasil and approved on 21 February 2019 by the Ethics Committee of Hospital Geral de Fortaleza, under protocol number 3.158.600.

## 3. Results

The study included 256 health professionals with a mean age of 39.4 years old. A total of 94.9% were female, 51.8% were married, and 64.9% had children who were financially dependent on them.

Regarding their level of education, 42% had graduated, 26.8% had completed higher education, and 31.2% had completed secondary/technical education. In relation to their professional category, 51.8% were nursing technicians, 22.7% were physicians, 19.1% were nurses, and 6.4% were physiotherapists or speech therapists. The majority had been working in an NICU for less than 10 years (58.9%). Additional characteristics are described in Table 1.

Furthermore, in relation to the health of these professionals, it was observed that 42.6% reported having a disease and 36.8% took medication. Alcohol consumption was reported by 30.3% and smoking only by 1.6% of them. Only 38.4% practiced some regular physical activity (Table 1). Their body mass index (BMI) was also evaluated, and it was observed that only 36.5% had a normal BMI, 38.5% were overweight, and 24.6% were obese.

### 3.1. Health Professionals’ Work Characteristics

Table 2 presents the health professionals’ characteristics related to their working environment. Of the 256 professionals who participated in this study, 9.4% belonged to Hospital 1, 40.2% to Hospital 2, 33.2% to Hospital 3, and 17.2% to Hospital 4, according to inclusion criteria previously described. It was observed that 42.6% of the professionals worked during the daytime period (7 a.m. to 7 p.m.), followed by 34.8% who worked the night shift (7 p.m. to 7 a.m.), 13.7% who worked in the morning (7 a.m. to 1 p.m.), and 9% who worked in the afternoon (1 p.m. to 7 p.m.). Considering their time spent commuting, 42.2% reported that they spent between 30 and 60 min commuting, and 38.2% spent less than 30 min commuting. About 63.2% of the participants were exclusively working in this NICU. Only 36 participants answered the question about the number of continuous working hours in the NICU; however, the majority of health professionals worked 12 or more uninterrupted hours at this workplace (83.3%) (Table 2).

### 3.2. Health Professionals’ Subjective and Objective Stress Levels

The participants’ self-perceptions of their physical state at the beginning of their work shift were also analyzed. From the 243 professionals who answered this question, 36.2% felt “rested”, 53.5% “a little tired”, and 10.3% “very tired”. At the end of the shift, 18.9% reported being “rested”, 45.4% “a little tired”, and 35.7% “very tired”. Concerning eventful occurrences during their work shifts, only 35.8% reported an eventful occurrence, and among these, 40% were severe cases, 18.6% were cardiac arrests requiring reanimation, 15.7% were only admissions to the NICU, 11.4% were patients needing intubation, and 14.3% were deaths; most of the participants classified these events as tiring (58.1%) but fewer participants classified their work shift as stressful (47.2%) (Table 2).

Although most the health professionals’ salivary cortisol levels measured at the beginning of their work shift were normal (80.4%), 19.6% demonstrated increased levels at the beginning of their work shift (Table 2). However, a smaller effect on their salivary cortisol levels at the end of their work shift was observed, since 96.5% of the participants had normal cortisol levels and only 3.5% presented increased levels (Table 2).

**Figure 1:**
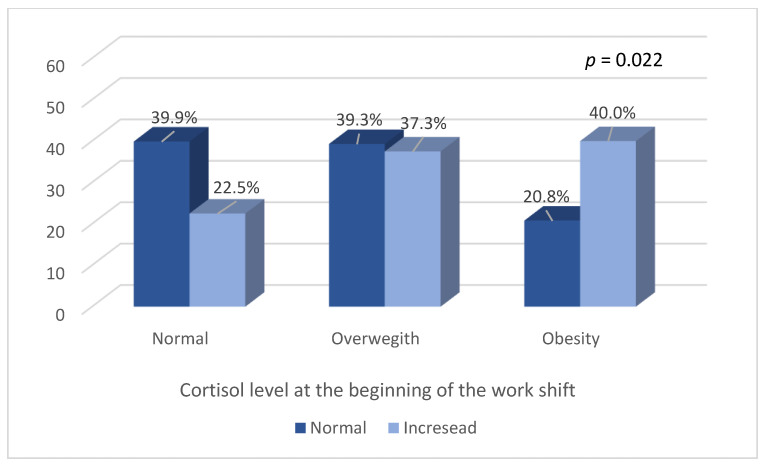
Body mass index and cortisol measurement at the beginning of the work shift.

**Figure 2:**
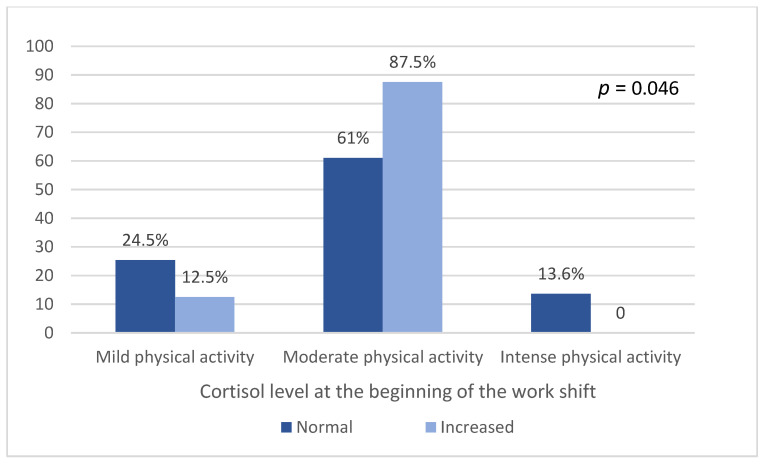
Type of physical activity and cortisol measurement at the beginning of the work shift.

A Chi-square test showed a statistically significant association between their salivary cortisol levels at the beginning of the work shift and their BMI (*p* = 0.022) (Figure 1) and type of physical activity (*p* = 0.046) (Figure 2). There was no association between salivary cortisol levels and physical activity practice (*p* = 0.515), alcohol consumption (*p* = 0.141), smoking habits (*p* = 0.58), the occurrence of diseases (*p* = 0.149), and the use of medications (*p* = 0.849). It can also be observed that among those who presented altered cortisol measurements at the beginning of their work shift, those who were classified as having a high BMI (overweight or obese) predominated (Figure 1).

**Figure 3:**
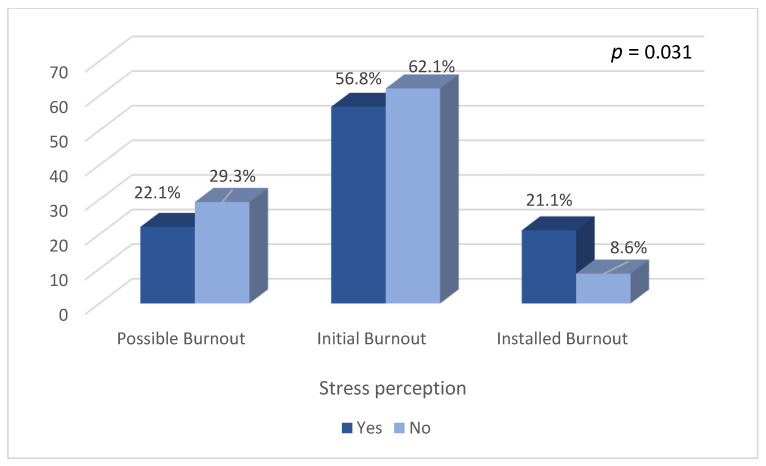
Perception of work shift as “stressful” and burnout phase.

Regarding the presence of burnout syndrome, of the 246 professionals who answered the questionnaire, 26% demonstrated “possible Burnout”, 58.9% were in the “initial Burnout”, and 15% demonstrated signs of “installed Burnout” (Table 2). A Chi-square test showed a statistically significant association between the occurrence of burnout syndrome and the perception of a stressful shift (*p* = 0.031) (Figure 3), which was not demonstrated in relation with other variables, such as initial physical state (*p* = 0.698), final physical state (*p* = 0.264), having a tiring shift (*p* = 0.230), and initial (*p* = 0.413) and final (*p* = 0.420) cortisol levels.

### 3.3. Health Professionals’ Chronotype and Associated Sociodemographic Factors

Most of the health professionals’ chronotypes belonged to the intermediate (46.7%) and morning (46.3%) types; only 7% of the participants were classified as evening type (Table 2). The results indicated that morning chronotype workers were significantly associated with the following: advanced age (*p* < 0.001), female gender (*p* = 0.032), married status (*p* = 0.014), and having children (*p* = 0.030) compared to those with evening and intermediate chronotypes (Table 3). In addition, significant differences were observed between the health professionals’ work experience according to their chronotypes (*p* = 0.025) (Table 3). However, there were no significant differences observed for the participants’ education level, type of position/function at the hospital, or number of children, according to their chronotype (Table 3).

### 3.4. Health Professionals’ Burnout Syndrome, Salivary Cortisol, and Chronotype

There was no association between the occurrence of signs of burnout syndrome and the participants’ chronotype (*p* = 0.316). Regarding their cortisol levels measured at the beginning of their shifts, health professionals presenting an intermediate-type chronotype demonstrated significantly increased cortisol levels than those of evening and morning workers (27.2% vs. 18.8% and 12.1%, respectively, *p* < 0.05) (Table 4). However, an association was not identified between their salivary cortisol at the end of their shifts and chronotype (*p* = 0.608) (Table 4). For their concentrations of cortisol at the beginning of their shift, the highest median found was in the intermediate group (0.21 µg/dL), and the median value of cortisol concentration at the end of their shifts was 0.07 µg/dL, regardless of chronotype. When observing the variation in cortisol between the initial and final periods and between the chronotype groups, it was identified that the intermediate type presented greater variation among others, although the difference was not significant (*p* = 0.153) when the Kruskal–Wallis’s test was applied. However, it is important to note that there was a reduction in the salivary cortisol levels from the beginning to the end of the shifts, showing a negative trend in each group (Table 4).

In addition, new groups were investigated for those who had chronotypes unfavorable to their periods of work, and their relationship with stress was evaluated. For this analysis, we considered a person with an “unfavorable chronotype” as someone whose work shift did not suit their chronotype. In the sample, it was identified that 87.7% had a favorable chronotype and 12.3% an unfavorable one. Among those who had a favorable chronotype, none of them scored as having “no signs of Burnout”, 25.8% scored as having a “possible Burnout”, 60.1% scored as in the “initial Burnout”, and 14.1% scored as “installed Burnout”. Among those who had an unfavorable chronotype, none of them scored as having “no signs of Burnout”, 31% scored as having a “possible Burnout”, 44.8% scored as in the “initial phase of Burnout”, and 24.1% scored as “installed Burnout”. However, there was no significant association between the unfavorable chronotype and the occurrence of burnout syndrome (*p* = 0.226) (Table 5).

On the other hand, a significant association was observed between having an unfavorable chronotype and altered cortisol levels at the beginning of the work shift (*p* = 0.006). It was observed that the group with an unfavorable chronotype arrived at the beginning of their shifts with a higher frequency of altered cortisol levels when compared to those of the group with a favorable chronotype (40% vs. 17%, *p* = 0.013) (Table 5). A significant association was also identified when analyzing the cortisol dosage at the beginning of the shift with the unfavorable chronotype, in which the median of the cortisol levels was increased in the group with an unfavorable chronotype in relation to those with a favorable chronotype (0.33 µg/dL vs. 0.18 µg/dL, *p* = 0.006) (Table 5).

## 4. Discussion

This study was designed to evaluate occupational stress and salivary cortisol levels in Brazilian health professionals working in NICUs and test for any evidence of an association with chronotype.

The profile of health professionals participating in our study was similar to that of several other scientific studies in terms of the following: the prevalence of nursing professionals, having a high degree level, having an average age around 40 years, and having worked for less than five years [1,43,44]. In Brazil historically the nursing profession is exercised by women [1]. This fact could explain the predominance of females in the sample. Therefore, they are also responsible for the management of daily activities in NICUs, team training, and patient care, putting them at risk of overload and psychological and chronic stress [47,48,49]. In addition to these factors, the intensive care environment, due to its structural and functional complexity with patients requiring great care and attention, is considered to be the hospital sector with the highest occurrence of occupational stress. This can lead to an increased risk of physical and mental illness [1,50,51], chronic stress, and burnout syndrome [2,52]. In our study, we did not find any participant with “no evidence of burnout” or “installed burnout”, which means that all the health professionals may be at risk for developing the syndrome to a greater or lesser extent, but most are in the “initial phase”, as published elsewhere [1,47,53].

The participants involved in our study were predominantly females with children, which, according to the literature, makes them more susceptible to the occurrence of burnout syndrome due to its correlation with long working hours and having to reconcile work with domestic tasks [54,55]. In fact, a Brazilian study identified factors such as working as a nurse or nursing technician, working night shifts, having a sedentary lifestyle, and having a sleep disorder as being associated with burnout syndrome in 84.8% of female health professionals who were working for less than 10 years [12,47,56], which is similar to most of our participants.

It was also observed a high percentage of professionals arrived at work already tired or very tired, and most of them considered their work shift as stressful as a result of the high demands related with typical work in an NICU. In these units, conflicts constantly arise, providing an environment of continuous stress, which slowly and almost imperceptibly spreads to professionals, leading to a delay in their perception of burnout syndrome symptoms [10].

Although the literature indicates that nursing professionals are among the most likely health professionals to present burnout syndrome (between 30 and 50%) [57], other workers can also be at risk, namely physicians (30 to 50%) [52,58], dentists (36.6%), and speech therapists (81.8%) [59].

Our participants also reported long working hours, namely more than 18 consecutive hours (52.8% of participants). The existing literature has estimated that working more than 12 consecutive hours and at night are associated with the occurrence of burnout syndrome [60], which was observed in 34.7% of our health professionals. In fact, working for consecutive hours may alter the functioning of the hypothalamic–pituitary–adrenal axis, and consequently, the production of cortisol, increasing stress [61,62]. In addition, if one’s work shift occurs at night, it can interfere with their biological circadian rhythms [8]; cause changes in sleep regulation and in turn, in the sleep–awake cycle; and change their levels of attention and concentration, which can lead to stress and affect the quality of the service provided due to cognitive and psychological fatigue [63,64].

Regarding chronotype, the intermediate type (46.7%) was the most prevalent, but it was lower than that found in the literature (60%) [34]. Our predominantly female sample showed a slight predominance between intermediate and morning types, in contrast to other studies, which identified the predominance of the morningness chronotype in women and the eveningness in men [31,65]. In addition, the participants’ chronotype was positively associated with age and having children, similar to other findings [66]. There was a prevalence of professionals with a favorable chronotype in relation to their work shift, as also found by Souza (2012), demonstrating a significant association between chronotype agreement and quality of life due to the alignment of individuals’ biological rhythms [39,67]. However, 12% of our participants demonstrated an unfavorable chronotype in relation to their work shift and a high incidence of burnout syndrome; however, we did not identify a significant association between having an unfavorable chronotype and the occurrence of burnout syndrome, in agreement with the literature [61].

Although normal salivary cortisol levels at the beginning of the shift were found in 70.7% of our health professionals and at the end of the shift were found in 86.7%, showing changes of 29.3% and 13.3%, respectively, these findings were lower than those observed in Argentinian health professionals (40.7% change) [52].

Furthermore, health professionals with an intermediate chronotype showed significant altered cortisol levels at the beginning of the shift (*p* < 0.05) when compared to their counterparts. In contrast to our expectations, the salivary cortisol levels significantly decreased from the beginning of the shift to its end (*p* < 0.05), which may be related with our participants’ professional experience and their capacity to cope with stress related with working in neonatal intensive care units. In fact, some studies in the literature have demonstrated that chronic stress leads to an increase in basal cortisol [22,35], which may also explain these findings in our participants, who showed signs of chronic stress. The misalignment between an individual’s physiology and biological rhythms reduces their capacity to work and concentrate, and increases their risk of accidents at work, and increases absenteeism due to mental and physical changes [62]. Therefore, adequate guidance is of utmost importance to prevent this situation and improve workers’ quality of life. In fact, alterations in the circadian rhythm caused by evening work and night work habits can be related to several diseases, such as breast cancer, neurovegetative disorders (reduced memory, cognition, and behavior), cardiac and metabolic disorders (Type 2 Diabetes) [64,66,68,69,70], and chronic diseases [71]. On the other hand, the alignment between chronotype and work performance suggests a protective factor for the quality of life of health professionals [67]. Therefore, the timings for one’s social and work commitments as well as daily habits should be adapted to each individual’s chronotype, avoiding a desynchronization of the circadian rhythm and the sleep–awake cycle [31,72].

## 5. Conclusions

The present study showed that health professionals working in NICUs were under a significant amount of stress during their work shifts and presented symptoms of burnout. Participants who carried out their work in disagreement with their chronotype showed increased salivary cortisol levels at the beginning of their work shift and the potential to develop burnout, in particular health professionals with the intermediate chronotype.

Therefore, circadian rhythm misalignment may interfere with stress in stressful work environments and compromise professionals’ health and capacity to work in an NICU. Special attention should be given to female workers, who normally have further domestic tasks, depending on their social and cultural environment. The adaptation of professionals’ working hours according to their biological rhythm is also recommended to avoid potential negative effects on their physical and mental health.

A limitation of this study was the small number of male participants in the sample, which made it challenging to conduct sex-based comparisons and other analyses. To address this limitation, further research is recommended with a larger sample of male and female participants to investigate the correlation between chronotype and stress in health professionals of both genders.

## Figures and Tables

**Table 1:** Sociodemographic characteristics and lifestyle habits of health professionals.

Parameter	Sample (*n* = 256)
Age, years (*n* = 251)	39.4 ± 9.8
Gender (*n* = 256)	
Male	13 (5.1)
Female	243 (94.9)
Body Mass Index (*n* = 244)	
Mild thinness	1 (0.4)
Normal	89 (36.5)
Overweight	94 (38.5)
Obese	60 (24.6)
Marital status (*n* = 251)	
Married	130 (51.8)
Stable union	21 (8.4)
Single	83 (33.1)
Divorced	17 (6.8)
Children (*n* = 251)	
Yes	163 (64.9)
No	88 (35.1)
How many children (*n* = 163)	
1 to 2	131 (80.4)
3 or more	32 (19.6)
Schooling (*n* = 251)	
Middle level	78 (31.1)
Higher level	67 (26.7)
Postgraduate	105 (41.8)
Function (*n* = 251)	
Doctor	57 (22.7)
Nurse	48 (19.1)
Nursing technician	130 (51.8)
Physiotherapist or Speech Therapist	16 (6.4)
Time working in NICU (*n* = 251)	
Less than 5 years	105 (41.8)
5 to 10 years	43 (17.1)
10 to 20 years	86 (34.3)
20 years or more	17 (6.8)
Disease (*n* = 251)	
Yes	107 (42.6)
No	144 (57.4)
Medication (*n* = 247)	
Yes	91 (36,8)
No	156 (63.2)
Smoking (*n* = 251)	
Yes	4 (1.6)
No	247 (98.4)
Alcohol (*n* = 251)	
Yes	76 (30.3)
No	175 (69.7)
Physical activity (*n* = 250)	
Yes	96 (38.4)
No	154 (61.6)
Intensity of physical activity (*n* = 84)	
Mild	121 (25)
Moderately	55 (65.5)
Intense	8 (9.5)

Categorical data expressed as absolute counts and percentages in parentheses. Quantitative data expressed as mean ± standard deviation. BMI: body mass index; NICU: neonatal intensive care unit.

**Table 2:** Characteristics of the professional situation related to the evaluated work environment.

Characteristics	Sample (*n* = 256)
Place (*n* = 256)	
Hospital 1	24 (9.4)
Hospital 2	103 (40.2)
Hospital 3	85 (33.2)
Hospital 4	44 (17.2)
Type and duration of work shift (*n* = 256)	
Morning—6 h	35 (13.7)
Afternoon—6 h	23 (9.0)
Daytime—12 h	109 (42.6)
Nighttime—12 h	89 (34.8)
Time spent commuting (min) (*n* = 249)	
<30	95 (38.2)
30–60	105 (42.2)
61 or more	49 (19.7)
Having another job (*n* = 250)	
Yes	92 (36.8)
No	158 (63.2)
Coming to work from another job (*n* = 251)	
Yes	40 (15.9)
No	211 (84.1)
Continuous hours of work (*n* = 36)	
6 h	6 (16.7)
12 h	11 (30.6)
18 h	8 (22.2)
24 h	11 (30.6)
Self-perception of physical state at the beginning of the shift (*n* = 243)	
Very tired	25 (10.3)
A little tired	130 (53.5)
Rested	88 (36.2)
Self-perception of physical state at the end of the shift (*n* = 227)	
Very tired	81 (35.7)
A little tired	103 (45.4)
Rested	43 (18.9)
Eventful occurrences during the shift (*n* = 232)	
Yes	83 (35.8)
No	149 (64.2)
Type of eventful occurrences during the shift (*n* = 70)	
Death	10 (14.3)
Reanimation	13 (18.6)
Admission	11 (15.7)
Intubation	8 (11.4)
Critical ones	28 (40)
Do you find the shift tiring? (*n* = 203)	
Yes	118 (58.1)
No	85 (41.9)
Do you find the shift stressful? (*n* = 197)	
Yes	93 (47.2)
No	104 (52.8)
Cortisol levels (µg/dL) at beginning of shift (*n* = 225)	0.19 (0.11–0.36)
Normal	181 (80.4)
Increased	44 (19.6)
Cortisol levels (µg/dL) at end of shift (*n* = 230)	0.07 (0.05–0.11)
Normal	222 (96.5)
Increased	8 (3.5)
Occurrence of Burnout Syndrome (*n* = 246)	
Possible occurence	64 (26)
Initial phase of Burnout	145 (59)
Installed Burnout	37 (15)
Chronotype (*n* = 244)	
Evening	17 (7)
Intermediate	114 (46.7)
Morning	113 (46.3)

Categorical data expressed as absolute counts and percentages in parentheses. Cortisol expressed as median and interquartile range in parentheses.

**Table 3:** Sociodemographic characteristics of professionals according to the chronotype classification.

	Chronotype Classification			
	Evening (*n* = 17)	Intermediate (*n* = 114)	Morning (*n* = 113)	*p*
Age (years)	36 ± 12	37 ± 9	43 ± 10	<0.001 ^a^
Gender				0.032
Male	3 (17.6)	7 (6.1)	3 (2.7)	
Female	14 (82.4)	107 (93.9)	110 (97.3)	
Marital Status				0.014
Married	6 (35.3)	52 (45.6)	69 (61.1)	
Stable union	2 (11.8)	9 (7.9)	10 (8.8)	
Single	5 (29.4)	46 (40.4)	29 (25.7)	
Divorced	4 (23.5)	7 (6.1)	5 (4.4)	
Have Children	8 (47.1)	65 (57)	83 (73.5)	0.030
Number of children				0.051
0	9 (52.9)	47 (42)	25 (22.9)	
1 to 2	7 (41.2)	54 (48.3)	64 (58.7)	
3 or more	1 (5.9)	11 (9.8)	20 (18.3)	
Financial Dependency	6 (35.3)	53 (46.9)	62 (56.4)	0.160
Education level				0.783
Postgraduate studies	8 (47.1)	43 (37.7)	52 (46.1)	
Higher	3 (17.7)	31 (27.2)	31 (27.5)	
High School/Technical	6 (35.3)	43 (34.2)	30 (26.6)	
Function				0.608
Doctor	3 (17.6)	23 (20.2)	30 (26.5)	
Nurse	4 (23.5)	21 (18.4)	21 (18.6)	
nursing technician	9 (52.9)	65 (57)	52 (46)	
Physiotherapist and Speech Therapist	1 (5.9)	5 (4.4)	10 (8.8)	
Work experience in NICU				0.025
Less than 5 years	8 (47.1)	56 (49.1)	38 (33.6)	
5 to 10 years	1 (5.9)	22 (19.3)	19 (16.8)	
10 to 20 years	6 (35.3)	34 (29.8)	43 (38.1)	
More than 20 years	2 (11.8)	2 (1.8)	13 (11.5)	

NICU: Neonatal intensive care units. Categorical data expressed as absolute counts and percentages in parentheses. Quantitative data were expressed as mean ± standard deviation. For quantitative data, the ANOVA test was used, and the Chi-square test for categorical data. ^a^ Significant differences were observed between morning type group vs. other chronotype groups using Tukey‘s post hoc test (*p* < 0.05).

**Table 4:** Health professionals’ signs of burnout syndrome and salivary cortisol, according to their chronotype.

	Chronotype Classification			
	Evening (*n* = 17)	Intermediate (*n* = 114)	Morning (*n* = 113)	*p*
Burnout rating ^a^				0.316
Possible Burnout	1 (5.9)	30 (26.3)	33 (29.7)	
Initial Burnout	12 (70.6)	68 (59.6)	61 (55)	
Installed Burnout	4 (23.5)	16 (14)	17 (15.3)	
Initial cortisol rating ^a^				0.027
Normal	13 (81.3)	75 (72.8)	87 (87.9)	
Changed	3 (18.8)	28 (27.2)	12 (12.1)	
Final cortisol rating ^a^				0.608
Normal	15 (93.8)	103 (97.2)	100 (98)	
Changed	1 (6.3)	3 (2.8)	2 (2)	
Cortisol dosage at the beginning of the shift (µg/dL) ^b^	0.19 (0.13–0.55)	0.21 (0.13–0.42)	0.16 (0.11–0.29)	0.069
Cortisol dosage at the end of the shift (µg/dL) ^b^	0.07 (0.05–0.12)	0.07 (0.05–0.12)	0.07 (0.05–0.1)	0.713
Cortisol variation during the shift (µg/dL) ^b^	−0.08 (−0.37–−0.03)	−0.13 (−0.34–−0.03)	−0.09 (−0.19–−0.03)	0.153

^a^ Categorical data expressed as absolute counts and percentages in parentheses. ^b^ Quantitative data expressed as median and interquartile range in parentheses. For quantitative data, the Kruskal–Wallis’s test was used, while the Chi-square test was applied for categorical data. Bold values represent the significant results (*p* < 0.05).

**Table 5:** Relationship of professional stress assessed by burnout and cortisol levels with the unfavorable chronotype of health professionals.

	Unfavorable Chronotype		*p*
	No (*n* = 214)	Yes (*n* = 30)	
Burnout Rating ^a^			0.226
Possible	55 (25.8)	9 (31)	
Initial Burnout	128 (60.1)	13 (44.8)	
Installed Burnout	30 (14.1)	7 (24.1)	
Physical state—Tired at the start? ^a^			0.761
Very	20 (9.8)	4 (14.3)	
A little	108 (52.7)	14 (50)	
Rested	77 (37.6)	10 (35.7)	
Physical state—Tired at the end? ^a^			0.410
Very	70 (37)	10 (35.7)	
A little	81 (42.9)	15 (53.6)	
Rested	38 (20.1)	3 (10.7)	
Eventful occurances on duty ^a^	73 (37.2)	9 (33.3)	0.693
Tiring duty	109 (57.4)	14 (53.8)	0.734
Stressful shift	86 (46.2)	9 (39.1)	0.518
Initial cortisol ^a^			0.013
Normal	160 (82.9)	15 (60)	
Changed	33 (17.1)	10 (40)	
Final cortisol rating ^a^			0.527
Normal	193 (97.5)	25 (96.2)	
Changed	5 (2.5)	1 (3.8)	
Cortisol dosage at the beginning of the shift (µg/dL) ^b^	0.18 (0.11–0.31)	0.33 (0.18–0.60)	0.006
Cortisol dosage at the end of the shift (µg/dL) ^b^	0.07 (0.05–0.11)	0.09 (0.05–0.13)	0.382
Cortisol variation during the shift (µg/dL) ^b^	−0.10 (−0.21–−0.03)	−0.20 (−0.48–−0.06)	0.06

^a^ Categorical data were expressed as absolute counts and percentages in parentheses. ^b^ Quantitative data were expressed as mean ± standard deviation. For quantitative data, the Mann–Whitney test was used, and the Chi-square test was used for categorical data. Bold values represent the significant results (*p* < 0.05).

## Data Availability

The authors confirm that the data supporting the findings of this study are available within the article.
